# Fisheries Inspection in Portuguese Waters from 2015 to 2023

**DOI:** 10.1038/s41597-024-03088-4

**Published:** 2024-04-10

**Authors:** Ricardo Moura, Nuno Pessanha Santos, Alexandra Vala, Leonor Mendes, Paula Simões, Miguel de Castro Neto, Victor Lobo

**Affiliations:** 1 Centro de Matemática e Aplicações (NovaMath), Universidade Nova de Lisboa, 2829-516 Caparica, Portugal; 2 Portuguese Navy Research Center (CINAV), Portuguese Naval Academy (Escola Naval), Almada, 2810-001 Portugal; 3 Department of Mathematics, ISEG - School of Economics and Management, Universidade de Lisboa, Lisboa, Portugal; 4 Portuguese Military Research Center (CINAMIL), Portuguese Military Academy (Academia Militar), Lisbon, 1169-203 Portugal; 5 Institute for Systems and Robotics (ISR), Instituto Superior Técnico (IST), Lisbon, 1049-001 Portugal; 6 NOVA Information Management School (Nova IMS), Universidade Nova de Lisboa, Lisbon, 1070-312 Portugal

**Keywords:** Biogeography, Water resources

## Abstract

As a coastal state, Portugal must ensure active surveillance over its maritime area, ensuring its proper control and inspection. One of the most critical inspection activities is the fishery inspection. To protect biodiversity, we must ensure that all the ships comply with the existing safety regulations and respect the current fishing quotas. This georeferenced dataset describes the fisheries inspections done in Portuguese waters between 2015 and 2023. Since we are dealing with occurrences that may have originated some legal process to the ship’s owner, we have ensured data anonymization by pre-processing the dataset to maintain its accuracy while guaranteeing no unique identifiers exist. All the pre-processing performed to ensure data consistency and accuracy is described in detail to allow a quick analysis and implementation of new algorithms. The data containing the results of these inspections can be easily analyzed to implement data mining algorithms that can efficiently retrieve more knowledge and, *e.g*., suggest new areas of actuation or new strategies.

## Background & Summary

As a coastal state, Portugal must ensure surveillance over its maritime area. The United Nations Convention on the Law of the Sea (UNCLOS) defines each coastal state’s territorial waters and Exclusive Economic Zone (EEZ) as generally extending to 200 nautical miles from shore^1^. According to this Convention, the coastal state can impose its laws and regulations, including fisheries, in its territorial waters extending up to 12 nautical miles from shore^2^. In the EEZ, each state has special rights regarding exploiting existing living natural resources, including regulating and managing the existing fishing quotas^3^.

In Portugal, the Navy performs most of the coast guard duties, and in particular, the Portuguese Navy (PoN) is responsible for most fishery inspections. The Portuguese Naval Command (PoNC) is the operational command responsible for managing and planning all PoN naval operations. This is important since the same institution gathers almost all the data needed for analysis, increasing the gathered data accuracy. To increase the dataset utility, consistency, and accuracy, all the data was also correlated with the existing records in the Portuguese Directorate-General for Natural Resources, Safety and Maritime Services (DGRM)^4^, the United Nations Code for Trade and Transport Locations (UN-LOCODE)^5^, and the European Union Fleet Register (EU-FR)^6^. The correlation between the datasets allowed for the creation of a highly comprehensive and distinctive dataset.

**Fig. 1:**
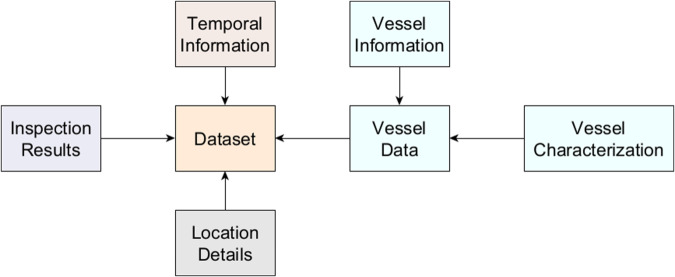
Simplified diagram of the dataset content.

The dataset describes 10,553 fishery inspections in Portuguese waters from 2015 to 2023. This data was collected from multiple sources, compared, and pre-processed to ensure accuracy. After pre-processing the data, we obtained a dataset that correctly characterizes the georeferenced fishery inspection occurrences in Portuguese waters. As will be described in detail in the *Data Records* section, the dataset contains the following information on each inspection (Fig. 1):

- **Temporal information -** Year, Month, Day, and Day period;
- **Location details -** Coordinates of the location where the vessel was inspected and the location where the Vessel has been registered with the inclusion of its associated Nomenclature of Territorial Units for Statistics - Level 2 (NUTS II) code;
- **Vessel information -** Sub-type of the commercial fishing vessel, observed fishing gear during the inspection and official main fishing gear in the registry, date of entry into service, and year of construction;
- **Vessel characterization -** Type of Vessel, dimensions, weight, power of engines, and hull material;
- **Result of Inspection -** Result of the inspection and recorded infractions.

An essential aspect of this dataset is the need to ensure data confidentiality and protection, especially considering that certain fishery inspections may have resulted in legal processes. The dataset has been carefully anonymized, thus avoiding disclosure of the actual ship involved by combining rounding of values and adding random noise. The disclosure protection methodology was implemented while preserving the overall quality of the dataset, as only selected features underwent slight transformations, while others remained unchanged. This approach ensures that direct matching of records to vessel identification is impossible, thereby safeguarding the privacy of the involved parties.

Maintaining a constant presence in all the maritime areas where Portugal has jurisdiction, even with a concept of operation based on a dual-use navy^7^ that conjugates defense and maritime authority resources, is impossible with the necessarily limited resources that exist. Due to this, analyzing the obtained fisheries inspection data is critical since it allows us to get essential knowledge that can help optimize human and material resources. The obtained results can also be easily generalized to other case studies since most coastal states present the same context of limited resources compared to their maritime area. This knowledge will help us to improve our decision-making process.

## Methods

The dataset was created considering an extensive analysis of the Fiscalization Reports (FISCREP) made during the PoN fishery inspections, which include information regarding vessel, fishing gear, documentation, *etc*., and the information retrieved from DGRM^4^, UN-LOCODE^5^, and EU-FR^6^. As happens in any dataset creation, extensive data pre-processing and validation were carried out to ensure the accuracy of the data. Some scripts were created using Python to make this pre-processing easier. All the adopted methods to guarantee the dataset accuracy, including all the data processing, will be described in this section in detail. Before the release and publication of the data, all the necessary measures of disclosure control were applied to avoid the possibility of direct identification of the inspected vessels, particularly the vessels that presumably committed an infraction.

**Fig. 2:**
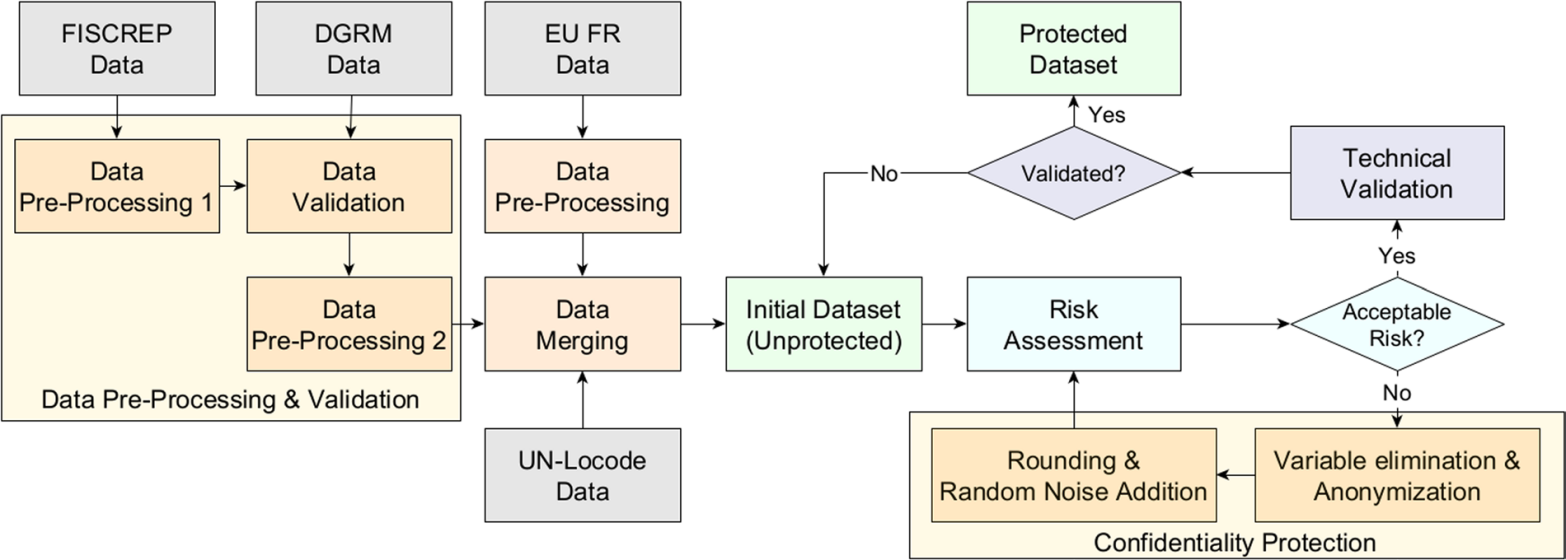
Simplified schematic of the approach used to create the initial dataset, perform confidentiality protection, risk assessment, and technical validation to obtain the final protected dataset.

Figure 2 outlines the process undertaken to create the protected dataset, which is detailed further in subsequent sections. Initially, the dataset undergoes data pre-processing and validation, utilizing information from various sources to compile the raw, unprotected dataset. The confidentiality protection will be ensured by two steps: Variable elimination & Anonymization and Rounding & Random noise addition. The first step involves carefully removing or modifying direct identifiers and sensitive information to prevent the risk of identifying individual vessels. The second step further masks the data, enhancing privacy without significantly compromising the data’s utility. After we ensure that we obtain an acceptable risk after implementing the confidentiality protection methods, a technical validation is performed to ensure that our final dataset has the needed quality to represent the unprotected dataset while preserving the majority of the integrity and utility of the data.

### Data acquisition

Data was acquired directly from multiple fisheries report sources, usually starting with the data contained in the FISCREP. Matching and comparing data from the different sources proved to be very useful and enabled us to obtain a complete data record to characterize each inspection correctly.

#### FISCREP data

The main data source was the PoNC since all the PoN-executed fishery inspections are reported in a standardized FISCREP form. A FISCREP is made every time a ship is inspected for fishing activities in waters under national sovereignty or jurisdiction. The ship, whether commercial, recreational, or for leisure fishing, may be either national or foreign. The FISCREP contains the information described in Table 1. To obtain the original fisheries inspection data, interested users must request it from the Portuguese Navy using the contact information provided on its website (https://www.marinha.pt). Users should provide the intended purpose for accessing the data when requesting. Once the request is approved for data sharing, a signed confidentiality agreement will be required to ensure that the data remains confidential and is not disclosed to others. The DGRM^4^ (https://www.dgrm.mm.gov.pt/frota) and European Union (EU) (https://webgate.ec.europa.eu/fleet-europa/search_en) data used in this study are publicly available on their respective websites.

**Table 1:** FISCREP data - Content description.

Registered Information	Description
***N***^***o***^	Inspection identification number assigned by the PoN
***Nome***	Vessel name
***CFR***	Community Fleet Register (CFR)
***n***^***o***^ ***reg***	Registration number of the vessel
***Latitude***	The latitude where the inspection took place
***Longitude***	The longitude where the inspection took place
***GDH***	Group-Date-Hour (GDH) or Date-Time Group (DTG)
***Unidade***	The PoN ship that conducted the inspection
***FIS***	A counter of the number of inspections carried out in a given period by the PoN ships
***Tip Emb***	The type of activity that the vessel is intended to perform
***Sub Tip***	The type of fishing gear allowed to be used
***Arte***	The fishing gear being used at the time of inspection
***Result***	The outcome of the inspection: “Legal vessel” or “Presumed violator”
***Infrac***	Infraction codes (for “Presumed violators”

The provided dataset included occurrences from January 2015 to February 2023. However, in its original form, the raw data contains unique identifiers that can easily identify the inspected ships. Thus, this data will later be anonymized, removing all unique identifiers while maintaining the dataset characteristics. After anonymization and confirmation that each individual entry no longer harbored the potential to directly identify any inspected vessel with a presumed infraction record, the final dataset^8^ received approval for public release and was subsequently made accessible to the broader community.

The original FISCREP-based dataset was created in Portuguese and then translated to English using a pre-processing script based on Python’s Pandas library to rename the existing labels. Due to confidentiality reasons, the original dataset and its translation cannot be made publicly available. However, the translation script is available at a GitHub repository (https://github.com/ricardomourarpm/Fishery_Inspection_PT_2017_23.git).

#### DGRM data

The publicly available DGRM data describes the registered fishing ships in 2021 and 2022. This information is helpful to complement and correct existing information about each fishing ship. The data is divided into two files, one for each year, containing the data described in Tables 2 and 3.

**Table 2:** DGRM data from 2021 - Columns description.

***Ano/Year***	Year in which the vessels were registered
***CFR***	Community Fleet Register Number
***Conjunto Identifica****ç****ão/Identification Set***	Vessel registration and name

**Table 3:** DGRM data from 2022 - Columns description.

***COD-REGIAO***	Geographic region code where the vessel is registered
***NUM_CFR***	Community Fleet Register number
***TXT_CONJ_IDENT***	Vessel registration and name
***NOM_EMBA_BASE***	Name of the vessel

#### EU-FR data

The EU provides a publicly available dataset describing all the ships authorized to perform fishing in EU waters. The respective ship flag country must constantly update this dataset to ensure continuous monitoring and control of its activities to preserve fish biodiversity.

The available information allowed us to obtain the ship’s CFR number from a reliable source and match it with its name and registration number. The CFR number is significant since it is a unique and common way of identifying a ship. Thanks to the existing Portugal-Spain bilateral agreements, it was also possible to retrieve information about the existing Spanish fishing ships and understand their fishing activities along Portuguese waters. The data can be easily retrieved online by selecting the active ships over a specific time interval. We have selected information regarding Portuguese and Spanish ships between January 2015 and March 2023. The obtained variables are described in Table 4. For more details about the Table 4 data content, check the Communication and Information Resource Centre for Administrations, Businesses and Citizens (CIRCABC) user interface^9^.

**Table 4:** EU FR data - Portugal and Spain registered fishing ships.

Column Name:	Description:
***Country of Registration***	Member state where the vessel is registered
***CFR***	Community Fleet Register Number (CFR)
***UVI***	Unique Vessel Identifier (UVI)
***Event***	Type of event recorded for the vessel, e.g., registration or license renewal
***Event Start Date***	Date when the event started
***Event End Date***	Date when the event ended
***Registration Number***	Vessel registration number given by the Member State
***External marking***	External marking of the vessel
***Name of vessel***	Name of the vessel
***Place (mainly Port) of registration***	Place where the vessel is registered
***IRCS***	International Radio Call Sign (IRCS) of the vessel
***IRCS indicator***	Indicator of whether the vessel has an IRCS
***License indicator***	Indicator of whether the vessel has a license
***VMS indicator***	Indicator of whether the vessel has a Vessel Monitoring System (VMS)
***ERS indicator***	Indicator of whether the vessel has an Electronic Reporting System (ERS)
***AIS indicator***	Indicator of whether the vessel has an Automatic Identification System (AIS)
***MMSI***	Maritime Mobile Service Identity (MMSI) number of the vessel
***Vessel Type***	Type of vessel according to the International Standard Statistical
	Classification of Fishery Vessels (ISSCFCG)
***Main fishing gear***	Main type of fishing gear used by the vessel according to the ISSCFCG
***Subsidiary fishing gear 1–5***	Types of subsidiary fishing gear used by the vessel according to the ISSCFCG
***LOA***	Length Overall of the vessel (meters)
***LBP***	Length Between Perpendiculars of the vessel (meters)
***Tonnage GT***	Gross Tonnage of the vessel
***Other Tonnage***	Other Tonnage of the vessel (Tonnes)
***GTs***	Gross Tons of the vessel
***Hull material***	Material used to build the hull of the vessel
***Power of Main Engine***	Power of the main engine of the vessel (kW)
***Power of Auxiliary Engine***	Power of the auxiliary engine of the vessel (kW)
***Date of entry into service***	Date when the vessel entered into service
***Segment***	Segment of the fleet to which the vessel belongs
***Country of importation/exportation***	Country where the vessel was imported/exported (if applicable)
***Type of export***	Type of export (if applicable)
***Public aid***	Vessel Public Aid Type
***Year of construction***	Ship year of construction

#### UN-LOCODE data

The publicly available data provided by the UN-LOCODE was used to enrich the dataset information and increase its accuracy. The UN-LOCODE data is publicly available on its website (https://service.unece.org/trade/locode/pt.htm). This process involved collecting (*web-scraping*) and cleaning the data, using Pythons Pandas library to obtain a table relating the location codes and their respective description and coordinates, as described in Table 5.

**Table 5:** UN-LOCODE data - Content description.

***Ch***	Character used to uniquely identify the location
***LOCODE***	Code used to uniquely identify the location in the United Nations Code
***Name***	Name of the location
***NameWoDiacritics***	Name of the location without diacritics
***SubDiv***	Subdivision of the country where the location is located
***Function***	Function of the location,e.g., commercial, military, or fishing
***Status***	Status of the location, e.g., open, closed, or under construction
***Date***	Date when the information about the location was last updated
***IATA***	International Air Transport Association (IATA) code for the location
***Coordinates***	Geographic coordinates (latitude and longitude) of the location
***Remarks***	Additional remarks about the location (if applicable)

### EU FR data - Pre-processing

At this stage, pre-processing is critical since we have several sources of information and must merge them to obtain an accurate final dataset^8^. The initial pre-processing was made according to the following:

1. Identification and sorting of the existing occurrences with the same *CFR* number;
2. Organization of the data according to the *Name of vessel* column;
3. Aggregation of the data generating lists of distinct *CFR* numbers, registration numbers, and places linked to each vessel name.

After pre-processing, the resulting data contains details regarding each distinct vessel name linked with its *CFR* numbers. We have the vessel’s name and a list of associated *CFR* numbers since certain names correspond to multiple vessels, registration numbers, and countries of registration (state flags). These attributes can be used to identify the inspected vessels accurately.

We have correlated the old and new denomination code letters with the location to identify possible changes in registration numbers and their registration codes. For instance, a previous Portuguese registration number might have been structured as *A-1111-Z*, while the new registration number for the same vessel could be *PTAVE-22222-Z*. *A* and *AVE* represent the section of the registration number associated with the place of registration. The unique *CFR* numbers and their precious and current denomination codes were also stored to maintain a record of the changes in registration numbers and location codes over time.

### FISCREP data - Pre-processing

While analyzing the FISCREP data, we obtained 43 records whose registration number was classified as *Unknown*. Since those records do not provide meaningful information, we removed them from the dataset. The main objective of this dataset is to focus on the commercial fishing vessels, only including those identified as *commercial fishing* in the final dataset^8^.

Most information gathered during an inspection is registered and inserted manually in the PoNC database. This means we will lack important information, especially the CFR number. To overcome this, we have performed a filtering process that analyzes the ships’ names with unique registration numbers. An iteration was performed for each unique vessel name. For each unique name, all associated *Registration Number*’s were grouped together, and the most frequent *Registration Number* was determined. This information was then recorded in a new database, enabling precise identification of the unique vessels based on the vessel name as the aggregation key for the records. This resulting dataset contains a separate list for each unique vessel name, storing all possible *Registration Number*’s, the most frequently occurring *registration number*, and the total count of *registration number*’s associated with that name.

The initial dataset consisted of 10,745 records of fishery inspections performed on commercial fishing vessels, with 3,643 records corresponding to a vessel name associated with only one registration number. To refine this dataset, we verify the 3,643 records to ensure that each vessel name is truly associated with a single registration number. We have also compared the remaining unchecked records, specifically using the registration number, with the datasets provided by DGRM. Through this comparison, we have identified the matching records, which helped us validate and verify the accuracy of the vessel information. After all these comparisons and rechecks, we had 1,093 records that required further validation and performed a new verification with the DGRM datasets from 2021 and 2022.

Since we have also considered the Spanish-registered vessels, we have also addressed those records. Considering that all Spanish-registered vessels within the European Union have registration numbers starting with 3, it was verified that most of the observed registration numbers did not conform to this pattern. Two alternative registration number columns were created to address this discrepancy and create a more consistent dataset. The first alternative registration number column was generated by adding 3 to registration numbers that did not begin with 3 or *PT* that correspond to the Portuguese vessel registration numbers. The second alternative registration number column was created to address cases where the original records contained a 3*-* prefix, which is an error since the European records did not include any hyphen. In those cases, the hyphen was removed to match the standard format of the European registration numbers. By introducing these alternative registration number columns, the dataset now provides additional options for identifying and cross-referencing vessels, ensuring consistency and compatibility with the standard registration numbering systems used by Spain and the European Union.

As mentioned, comparing the alternative registration numbers allowed further dataset refinement, increasing its accuracy. As a direct result, the number of unchecked inspected vessel records has been reduced to 985. Analyzing those 985 records, we can state that they correspond to 370 unique vessels whose registration numbers do not match existing records. Those inexistences are mainly justified by insertion or omission errors that may have occurred during the fishery inspection.

Since we cannot compare the existing records, we have extracted the unique names and registration numbers from the remaining unchecked records. These unique names were then stored with corresponding lists of associated registration numbers. We selected only those with a name associated with a single registration number from the previously checked records. With this process in place, the number of unchecked records has now been reduced to 537. The focus will be verifying the vessel names’ uniqueness, correctness, and associated registration numbers.

#### Vessels selection - Levenshtein distance

A similarity calculation was performed on the registration numbers associated with each vessel name to address the issue of misspelled vessel names in the unique records. This calculation aimed to determine which records might belong to the same vessel, allowing for the identification of misspelled or similar registrations. Calculating the similarity between registration numbers makes identifying potential matches and discrepancies possible. This process helps distinguish between genuine variations in registration numbers and instances where misspellings or errors occurred during the registration process.

The *Levenshtein* distance^10^ was implemented using Python to quantify the minimum number of single-character edits required to transform one string into another. For two strings, denoted as *s* and *t*, the *Levenshtein* distance (*lev*_*s,t*_) can be recursively calculated as follows^11^:

1 $$le{v}_{s,t}=\left\{\begin{array}{ll}\max \left(| s| ,| t| \right) & ,\,{\rm{if}}\,\min \left(| s| ,| t| \right)=0\\ \min \left\{\begin{array}{l}le{v}_{s-1,t}+1\\ le{v}_{s,t-1}+1\\ le{v}_{s-1,t-1}+{1}_{\left({s}_{i}\ne {t}_{j}\right)}\end{array}\right. & ,\,{\rm{otherwise}}\end{array}\right.$$

where |*s*| and |*t*| represent, respectively, the lengths of the strings *s* and *t*, ${1}_{\left({s}_{i}\ne {t}_{j}\right)}$ denotes the indicator function, which equals 1 if the *i*-th character of string *s* is not equal to the *j*-th character of string *t*, and 0 otherwise.

The final similarity score *similarity*_*s,t*_ is obtained by dividing the sum of the lengths of strings *s* and *t* minus the *Levenshtein* distance (*lev*_*s, t*_) by the sum of the lengths of strings *s* and *t*. The resulting score is then rounded up to the nearest integer. The formula for calculating the similarity score is as follows:

2 $$similarit{y}_{s,t}=\frac{| s| +| t| -le{v}_{s,t}}{| s| +| t| }.$$

To help the decision-making process, only the maximum and minimum similarity scores between each pair of registration numbers, denoted as *s* and *t* (where *s* ≠ *t*), were stored for each unique vessel name. However, since most of the remaining unchecked records are from Spanish vessels and their registration numbers typically start with 3, further steps were taken to address this. The alternative registration number where a *3* was added at the beginning was considered to find matching CFR records in the dataset. The similarity between these alternative registration numbers and the existing CFR records was computed. Specifically, a similarity score of 85% or higher was used as a threshold to determine matches between the alternative registration numbers and the CFR records. If a match was found, the new registration number was stored for the corresponding vessel name in the unchecked records.

In the final dataset^8^, each unique vessel name is associated with the following attributes:

- **List of registration numbers -** Contains all the registration numbers attributed to the vessel name;
- **Most frequent registration number -**: Represents the registration number that occurs most frequently among the records associated with the vessel name;
- **Number of registration numbers -** Indicates the count of registration numbers attributed to the vessel name;
- **Alternative registration number -** A modified registration number where *3* is added at the beginning of the string;
- **List of similarity scores -** Stores the similarity scores calculated between the alternative registration number and the CFR records;
- **Maximum & Minimum similarity scores -** Represents the highest and lowest similarity scores from the list of similarity scores;
- **Maximum similarity score with most frequent registration number -** Indicates the highest similarity score between the most frequent registration number and all the records in the EU FR data;
- **List of similar CFR records -** Contains the CFR records with a similarity score greater than 85% with the most frequent registration number and their corresponding recorded names.

The pre-processed dataset serves as a foundation for accurate manual decision-making processes. With careful inspection, cross-referencing with different sources, and considering the similarity measures, 105 vessels were correctly identified or selected as unique, and their names and registration numbers were corrected accordingly. The CFR column in the checked dataset was updated with the correct CFR information for those vessels where the corresponding CFR records were found. However, the CFR column was populated with the description *No_CFR* for vessels with no corresponding CFR records. This meticulous manual selection process ensures the accuracy and integrity of the dataset, rectifying any errors in vessel names and registration numbers and incorporating available EU FR data where possible.

#### FISCREP & EU FR data - Merging

After completing the pre-processing steps, the FISCREP, EU CFR, and DGRM datasets were merged based on each vessel registration number as the common identifier. This allowed the association of at least the CFR records with vessels not present in the EU FR data. Although these inspected vessel records may contain only some of the details in the EU FR data, they still provide valuable information regarding the location and type of infraction.

To focus on capturing the characteristics of each vessel in the final dataset^8^, certain variables from the EU FR data were considered less important (Table 4). The discarded variables were:

- *Event;*
- *Event Start Date;*
- *Event End Date;*
- *External marking;*
- *Name of vessel;*
- *IRCS;*
- *IRCS indicator;*
- *License indicator;*
- *VMS indicator;*
- *ERS indicator;*
- *AIS indicator;*
- *MMSI;*
- *GTs;*
- *Country_imp_exp.*

By excluding these variables, the main dataset focus remains on the critical characteristics of each vessel within the dataset. This approach ensures clarity and relevance in capturing the necessary information while optimizing the dataset for analysis.

#### UN-Locode data - Merging

We have also incorporated the local codes for the vessel registration locations to enhance the final dataset^8^ accuracy. The aim was to include a column with the corresponding new Portuguese code, which always starts with *PT*. Additionally, another column was added to include the names of the respective locations without diacritics, extracted from the UN-LOCODE data that was *web-scraped* earlier. Notably, these names were added specifically for the Portuguese locations, while others may not have this information. By merging this data, we provide a more comprehensive and standardized representation of the vessel registration locations within the dataset.

#### Final merged data - Pre-processing

To obtain the final complete dataset, several variables of interest were created, such as indicator variables for the type of infractions, the count of infractions per inspected vessel, and the NUTS II code representing the region where the vessel was registered. These additional variables provide valuable insights for better analysis and enrich the dataset with essential data. However, some variables were deemed not necessary and were subsequently removed from the dataset for the following reasons:

- ***Vessel_Type_x*** **-** Since we focused on commercial fishing vessels, this variable has only one value;
- ***FISCREP’s CFR*** **-** Due to limited recording during the inspection process, the CFR variable had low data availability and was not considered significant for analysis;
- ***FIS*** **-** The FIS variable was considered unimportant and thus removed from the dataset;
- ***Place of Registration*** **-** As most vessels in the dataset are from Portugal, the Place of Registration variable was deemed less important and removed;
- ***UVI*** **-** Another type of identification variable was removed from the dataset as it was not deemed significant for analysis;
- ***Event***, ***Event Start Date***, **and** ***Event End Date*** - These variables are related to the type of event registered in the European Fleet and were deemed not important for the analysis at hand;
- ***External Marking*** **-** As the External Marking variable was consistently equal to the Registration Number variable, it was redundant and removed from the dataset;
- ***Name of Vessel*** **-** In cases where the vessel name changed within the same boat or was a repetition of the Name variable, the Name of Vessel variable was considered less important and thus excluded;
- ***Country of Importation/Exportation*** **and** ***Type of Export*** **-** These variables had limited data availability, with a high proportion of missing values, and were therefore removed from the dataset.

Removing these less important variables makes the dataset more focused, streamlined, and suitable for the intended analysis. This allows for more precise insights and more efficient modeling processes.

### Confidentiality Protection

It is crucial to handle sensitive information appropriately when dealing with privacy and data protection. Confidentiality protection measures must be taken to maintain accuracy and ensure that unique identifiers do not exist. The next sections will explore all the techniques adopted to ensure confidentiality protection and risk assessment.

#### Variable elimination & Anonymization

In the obtained dataset, certain variables, referred to as Direct Identifiers^12,13^, directly reveal an individual’s identity without any ambiguity. These variables, including the registration number, CFR number, name of the inspecting unit, and the MMSI, can directly identify the inspected vessels and naval units involved in the inspections. Removing these direct identifier variables from the dataset is essential to ensure the privacy and anonymity of the individuals involved. However, only the anonymized CFR number and the anonymized unit will be retained to maintain the necessary information for analysis purposes.

We need to consider variables in the dataset that may lead to vessel identification in addition to direct identifiers. These variables, known as key variables^12,13^, possess characteristics that, when analyzed together, can potentially reveal the vessel’s identity. The vessel’s name and the *IRCS* are particularly significant among these key variables. When coupled with other information in the dataset, these variables can potentially expose the vessels’ identity.

The following variables were considered for removal or transformation from the dataset to be able to ensure data anonymization:

- ***GDH*** **-** The exact time of inspection, when combined with the location of the inspection, has the potential to be crosschecked online, leading to the identification of the vessel. Since other time variables are present in the dataset, this specific variable can be eliminated to mitigate the risk of identification;
- ***Location*** **-** As previously discussed, the location variables are crucial for analysis and will be retained in the dataset;
- ***IRCS indicator, License indicator, VMS indicator, ERS indicator****,* **and** ***AIS indicator*** **-** These binary variables, when combined with other variables, can identify certain vessels. For example, their combination with the *Main fishing gear* variable may lead to identifying 29 vessels. Due to the potential risk of identification and low importance, it was decided to remove these variables from the dataset;
- ***Date of entry into service*** **and** ***Year of construction*** **-** These variables possess unique qualities that may lead to identification. To mitigate this risk, it was decided to retain only the year’s information;
- ***Subsidiary fishing gears 1–5*** **-** Due to the high number of missing values and the potential identification risk when combined, these variables were removed from the dataset, as the *Main fishing gear* variable provides sufficient and more important information;
- ***LOA, LBP, Tonnage GT, Other tonnage, Power of main engine*, and** ***Power of auxiliary engine*** **-** These variables have many unique values and require transformation due to their precision;
- ***GTs*** **-** The high number of non-existent values in this variable represents a significant risk, and as a result, it will be removed from the dataset.

To ensure the anonymization of direct identifier variables, the following approach was implemented:

- **CFR Variable -** The CFR variable will be transformed into two different values:
- **CFR_#** - For vessels with an available CFR number. The actual CFR number was replaced with *CFR*_#, where # represents a unique number assigned to each vessel. This allows the analysis of vessels with known CFR numbers, potentially identifying repeated offenders and assessing inspection frequency;
- **NOCFR_#** - For vessels without a known CFR number. In this case, a variable was assigned to the value *NOCFR*_#, where # represents a unique number assigned to each vessel.
- **Unit Variable** - Each unit record will be associated with an anonymized code, *Unit_#*, where it will be assigned a unique number #, allowing for the identification and analysis of individual units while maintaining their anonymity.

### Rounding & Random Noise Addition

Two different risk metrics^14^ were employed to assess the risk associated with the key variables and explore the possibility of identifying new ones. The adopted risk metrics were:

- **Sample Frequency Count** - Measures the frequency count (*f*_*k*_) of the *k*-th key, which represents the combination of *k* key variables, within the FISCREP dataset. It provides insights into how often certain combinations of key variables occur within the sample;
- **Population Frequency Count** - Measures the frequency count (*F*_*k*_) of the *k*-th key, which represents the combination of *k* key variables within the FISCREP dataset. It provides insights into how often certain combinations of key variables occur within the sample.

The main objective is to identify situations where both the sample frequency count (*f*_*k*_) and the population frequency count (*F*_*k*_) are equal to 1 (*f*_*k*_ = *F*_*k*_ = 1). This scenario indicates a unique record in both the sample and the population, which poses the highest disclosure risk. These metrics were considered in the application of disclosure techniques. Once the disclosure process is complete, the metrics will be recalculated for combinations of up to four variables. This approach allows for the evaluation of disclosure risk and helps identify any potential combinations of key variables that may pose a risk of re-identification.

Regarding the variables *Date of entry into service* and *Year of construction*, it is acknowledged that they still possess key characteristics when reported only by the year. To ensure confidentiality, a rounding technique^12^ can be applied to the *Year of construction* by rounding it up to the nearest tens. Similarly, the same technique can be applied to the *Date of entry into service* by rounding it to the nearest year. However, it is noted that even after using the rounding technique, there are still 13 records (for *k* = 3) where the combination of the variables *Main fishing gear*, *Date of entry into service*, and *Year of construction* may lead to potential re-identification risks. To further enhance confidentiality, it was added random noise^15,16^ to these two variables according to:

3 $${x}_{i}^{* }={x}_{i}+{\varepsilon }_{i}$$

where *x*_*i*_ represents the original value, ${x}_{i}^{* }$ represents the new value, and ${\varepsilon }_{i}\mathop{ \sim }\limits^{{\rm{i}}{\rm{.i}}{\rm{.d}}{\rm{.}}}N(0,{\sigma }^{* })$. Here, σ^*^ is calculated as σ* = *ασ*_*x*_, where *σ*_*x*_ is the standard deviation of the original variable data. This noise addition process is performed for each of the 10,553 records in the dataset, resulting in new values ${x}_{i}^{* }$. To enhance privacy protection, the disclosed value in the FISCREP data will be the rounded version of ${x}_{i}^{* }$ to the nearest integer. Using this technique, the original data is distorted randomly, ensuring the new values are not significantly far from the real ones. The standard deviation is set as a proportion of the original variable’s standard deviation, and the parameter *α* will be disclosed to the public. This approach protects privacy while allowing meaningful analysis and insights from the modified dataset.

After careful consideration of the various variable combinations, it was determined that the variables *LOA*, *LBP*, *Tonnage GT*, *Other tonnage*, *GTs*, *Power of main engine*, and *Power of auxiliary engine* pose a potential risk of vessel identification. To address this concern, these variables underwent specific transformations to protect the privacy and confidentiality of the dataset.

Specific transformations were applied to address the identified risk associated with the variables *Other tonnage* and *Power of auxiliary engine*. The *Other tonnage* variable was rounded to the nearest integer, while the *Power of auxiliary engine* variable was rounded to the nearest tens. This rounding process can also be viewed as a form of global recoding, where the values of the variables are adjusted to fall within an interval with a range of 1 or 10, respectively. The result of the rounding process determines the center of this interval.

An alternative transformation was applied for the *Power of main engine* variable case due to the high risk of vessel identification even after rounding it to the nearest tens. Instead of rounding it to the nearest ten, random noise was added to the variable using Eq. 3. Additionally, in cases where the transformation resulted in a zero value, it was converted to the minimum value of the original *Power of main engine* variable. Furthermore, its absolute value was assumed if the transformed value was negative. A similar procedure addressed the identification risk associated with the *Tonnage GT* variable. Random noise was added to the variable also using Eq. 3.

For the variables *LOA* and *LBP*, simply performing a data rounding to the nearest integer was insufficient. An additive noise was introduced to the values using Eq. 3, rounding to the nearest decimal place to address this issue. During this transformation, only the absolute values were considered. Additionally, as all the resulting transformed values were non-zero, any instances of zero values were replaced with the minimum recorded value for each respective variable.

After completing the transformations, a local suppression technique was applied to four vessels for a categorical variable identifying them. This means the specific identifying information for these vessels was removed from the dataset to protect their confidentiality further. Additionally, a partial imputation technique was used to replace a specific variable value that posed a 100% risk of identification. The imputed value was determined as the average of that variable’s three closest unique values. It is worth noting that this imputation was only necessary for two vessels among all the presumed infractors.

Certain modifications were made to further protect against the risk of vessel identification based on the combination of the *GDH* variable and geographical coordinates. The inspection time was removed, and only the year, month, day, and period of the day were retained. The period of the day was divided into four categories: 0 h to 5 h59, 6 h to 11 h59, 12 h to 17 h59, and 18 h to 23 h59.

**Fig. 3:**
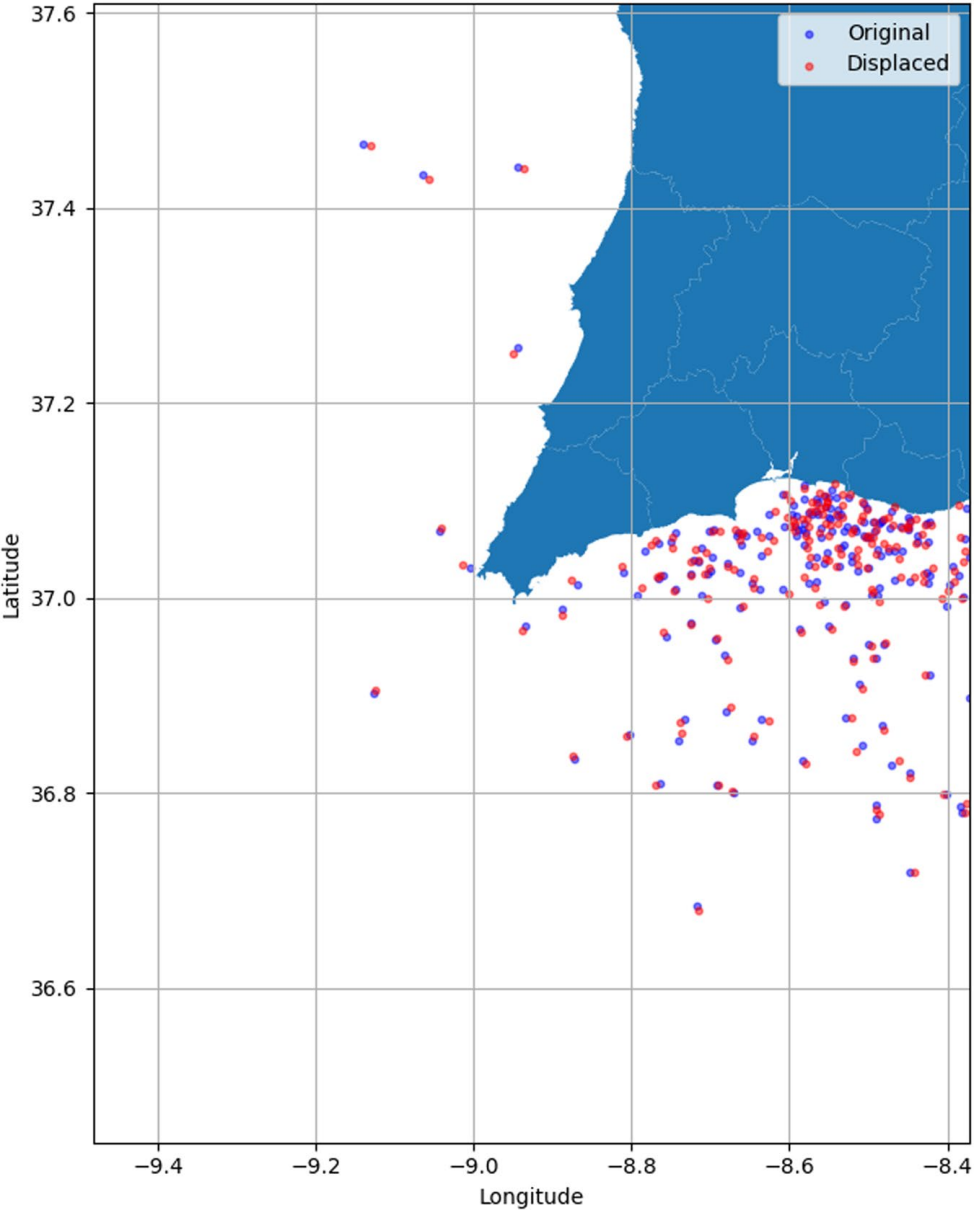
Original vs. Displaced coordinates - Illustration.

Additionally, a geographic displacement procedure^17^ was applied to introduce a random shift, seen as a random perturbation, in the latitude and longitude coordinates. The displacement values ranged from 0 to 0.5 nautical miles. The new value will be a random localization in the 1-nautical mile rectangle whose center is the real location. To illustrate, a selected set of records near the Sagres region in Portugal was included in Fig. 3 to demonstrate the final geographical displacement along with the original location.

By implementing these measures, the dataset is further safeguarded against potential vessel identification risks while preserving the integrity of the data for analysis purposes.

#### Risk Assessment

After completing all the variable transformations, a comprehensive risk assessment was conducted, considering all publicly available EU FR data variables. It was determined that the only sensitive variables in the dataset are those related to the presence of a presumable infraction and the type of infraction. Additionally, records with a result of *LEGAL* are considered non-sensitive.

Given this assessment, the focus was placed on the vessels with at least one infraction record. Among these vessels, only eight could potentially be identified. However, it is essential to note that even if these eight vessels are identified, the intruder cannot be sure of the accuracy of the identification due to the perturbation caused by the noise addition.

Only the variables that were left untouched due to their importance and those that underwent rounding transformations were considered to refine the risk assessment further. Using these selected variables, the identification of only two vessels was possible. However, it is essential to mention that these two vessels are no longer directly identifiable due to the local imputation replacement process that was previously described. Thus, the released data do not possess that risk anymore. No vessels can be identified as unique based on publicly available data. This means that each vessel in the dataset is indistinguishable from others regarding key variables (up to four combinations).

In conclusion, the final dataset^8^ ensures that no individual entry can directly identify an inspected vessel that presumably had an infraction record. This is the most crucial aspect to address when considering privacy protection. By achieving this level of anonymity, the dataset provides a good level of confidentiality and safeguards the identities of the inspected vessels, ensuring compliance with privacy regulations and standards.

## Data Records

The final released dataset^8^ consists of data pre-processed from multiple sources, encompassing various variables with diverse types of information. The dataset comprises 10,553 inspections, 8,917 inspections without infractions, and 1,636 records of vessels that presumably committed infractions. The 41 dataset variables can be described as follows:

- ***Latitude*** - The latitude coordinate of the inspected vessel’s location;
- ***Longitude*** - The longitude coordinate of the inspected vessel’s location;
- ***Unit*** - Anonymized code representing the inspecting naval unit (29 units);
- ***Sub_Type*** - The sub-type of the vessel registered during inspection (15 sub-types). The existing sub-types are: *Trawl*, *Traps*, *Multipurpose*, *Gillnet/trammel net*, *Longline*, *Towed Dredge*, *Line fishing vessel*, *Other*, *Seine net*, *Pole and line*, *Support vessel*, *Seaweed harvesting*, *Unknown*, *Factory vessel*, and *Unidentified*;
- ***Gear*** - Registered Fishing Gear of the vessel during inspection (24 fishing gears). The different values present in the *Gear* column are: *Trawl*, *Traps*, *Gillnetting*, *Trammel nets*, *All*, *Longline*, *Towed Dredge*, *Handline*, *Pots*, *Gear nei*, *Purse seine*, *Lines*, *Mixed gears*, *Traps bucket*, *Dropline*, *Pound nets*, *Scotish seines*, *Lift nets*, *Danish seines*, *Hand Dredges*, *Harpoon*, *Lampara nets*, and *Seines*;
- ***Result*** - The result of the inspection. A *LEGAL* value indicates that no infractions were detected, and *PRESUM* indicates that during the inspection, it was recorded at least one infraction;
- ***Infraction*** - The type of infraction committed by the vessel (categories I to XIV). The description of the Infractions is as follows^18^:
  - **I**: Non-existent logbook;
  - **II**: Incorrectly filled logbook;
  - **III**: Prohibited fishing gear;
  - **IV**: Fishing in a prohibited or restricted area;
  - **V**: Fishing prohibited due to excessive engine power or tonnage;
  - **VI**: Improper catches due to prohibited fishing;
  - **VII**: Improper catches due to bycatch;
  - **VIII**: Improper catches of undersized fish;
  - **IX**: Activity conducted without a license or authorization;
  - **X**: Improper marking or identification of fishing gear;
  - **XI**: Improper marking or identification of the vessel;
  - **XII**: Miscellaneous: Invalid certificates;
  - **XIII**: Miscellaneous: Non-existent/invalid maritime registration;
  - **XIV**: Miscellaneous: E.g., lack of onboard documents, lack of pyrotechnics, expired life-saving equipment, expired fire extinguishers, among others.
- CFR - Anonymized CFR number associated with the vessel (2285 vessels);
- Vessel_Type - The registration of the vessel type in the EU FR data according to ISSCFV (11 types). The different present categories are:
  - **DB**: Using boat dredge;
  - **FX**: Fishing vessels not specified;
  - **LL**: Longliners;
  - **LP**: Pole and line vessels;
  - **MO**: Multipurpose vessels;
  - **MOX**: Multipurpose vessels nei;
  - **TTW**: Tugboat/towboat;
  - **TTP**: Trawling trawlers;
  - **TU**: Tuna boats;
  - **WOX**: Workboats;
  - **SP**: Purse seiners.
- ***Main fishing gear*** - The registration of the primary fishing gear used by the vessel in the EU FR data according to ISSCFV (10 types).
  - **DRB**: Towed dredges;
  - **FPO**: Pots;
  - **GNS**: Set gillnets (anchored);
  - **GTR**: Trammel nets;
  - **LHP**: Handlines and hand-operated pole-and-lines;
  - **LLD**: Drifting longlines;
  - **LLS**: Set longlines;
  - **OTB**: Single boat bottom otter trawls;
  - **PS**: Purse seines;
  - **TBB**: Beam trawls.

- LOA* - Length overall of the vessel with added random noise following Eq. 3 and rounded to the nearest tenths (meters);
- LBP* - Length between perpendiculars of the vessel with added random noise following Eq. 3 and rounded to the nearest tenths (meters);
- Tonnage GT* - Gross tonnage of the vessel with added random noise following Eq. 3 rounded to the nearest tenths;
- Other tonnage* - Other tonnage information related to the vessel, rounded to the nearest integer (Tonnes);
- Power of main engine* - The power of the main engine of the vessel with added random noise following Eq. 3 rounded to the nearest tenths (kW);
- Power of auxiliary engine* - The power of the auxiliary engine of the vessel rounded to the nearest tens (kW);
- ***Hull material*** - The material used for the hull of the vessel. The values present in the dataset are:
  - **1**: Wood;
  - **2**: Metal;
  - **3**: Fiberglass/Plastic;
  - **4**: Other;
  - **6**: Polyester.

- Date of entry into service* - The date when the vessel entered into service with added random noise following Eq. 3 rounded to the nearest integer;
- Year of construction* - The year when the vessel was constructed with added random noise following Eq. 3 rounded to the nearest integer;
- ***real_local*** - Code associated with the vessel’s registration gathered from the Registration Number;
- ***Local_Name*** - The name of the location where the vessel is registered (without diacritics) only for the Portuguese vessels (44 locations);
- ***I–XIV*** **variables** - Indicator variables for different types of infractions (categories I to XIV);
- ***number_infracs*** **-**: The number of infractions registered for the vessel;
- ***Year*** - The year of the inspection;
- ***Month*** - The month of the inspection;
- ***Day*** - The day of the inspection;
- ***Period*** - The period of the day of the inspection (1 - From 0:00 to 5:59; 2 - From 6:00 to 11:59; 3 - From 12:00 to 17:59; 4 - From 18:00 to 23:59);
- ***NUTS II_Code*** - NUTS II code represents the region where the vessel is registered only for Portuguese vessels.

**Fig. 4:**
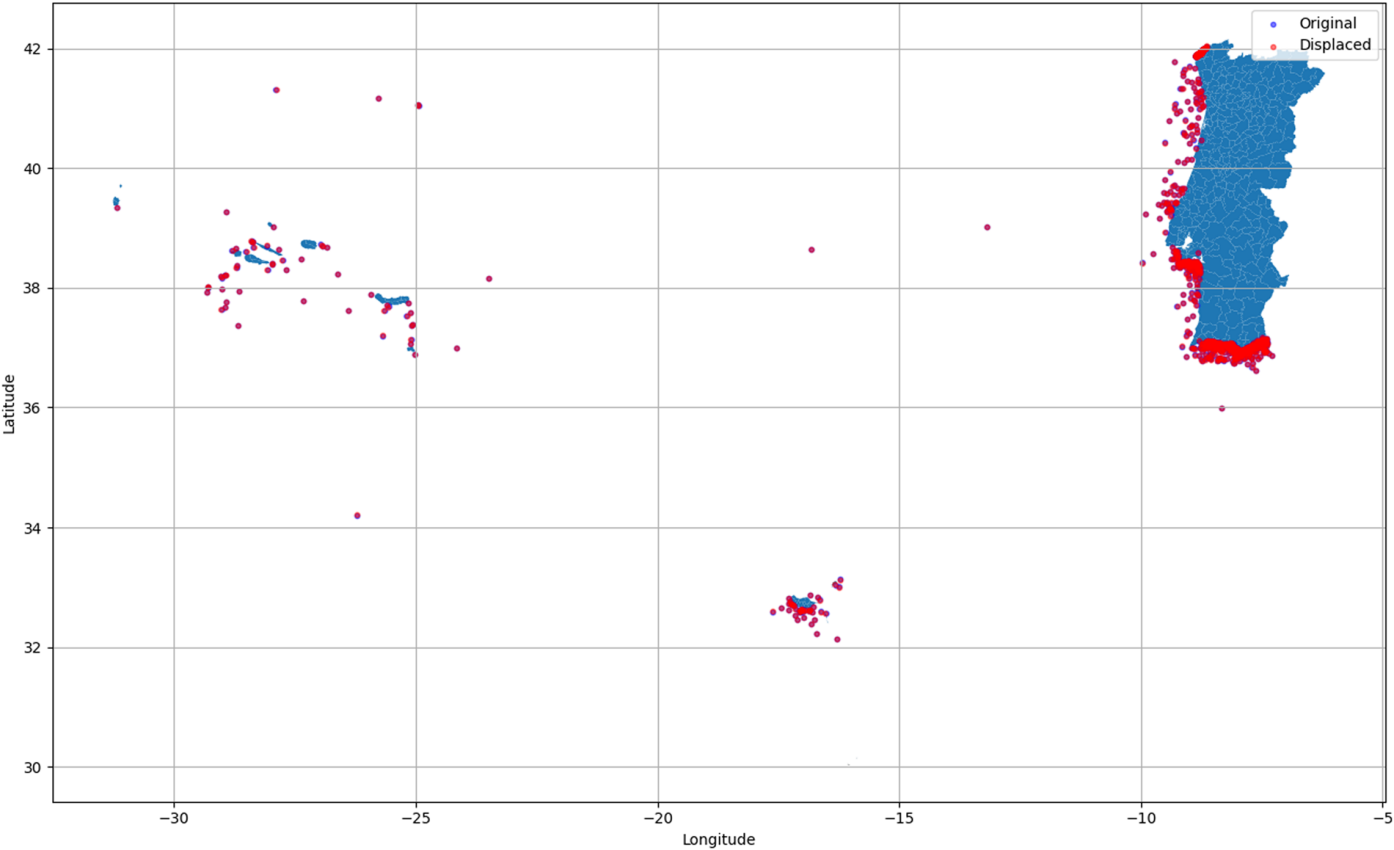
Dataset Real vs. Displaced coordinates representation.

You can observe a summary of these variables, along with brief descriptions and their respective data types, in Table 6. All the data variables marked with an asterisk (*) have undergone a transformation process to protect the identity of the inspected vessels, as explained in the previous Section. This transformation ensures that no vessel, particularly those with previous infraction records, can be directly identified by exactly matching key variables that are publicly available. Figure 4 provides a visual overview, demonstrating the overlapping positions of the displaced and real locations. Due to the scale adopted, it is impossible to distinguish deliberately between the two sets of locations.

**Table 6:** Description of the Dataset Variables - All the data variables marked with an asterisk (*) have undergone a transformation process to protect the identity of the inspected vessels.

Variable	Description	Type
*Latitude*	The latitude coordinate of the inspected vessel’s location	Text
*Longitude*	The longitude coordinate of the inspected vessel’s location	Text
*Unit*	Anonymized code representing the inspecting naval unit	Categorical
*Sub_Type*	The sub-type of the vessel registered during inspection	Categorical
*Gear*	Registered Fishing Gear of the vessel during inspection	Categorical
*Result*	The result of the inspection	Categorical
*Infraction*	The type of infraction committed by the vessel	Categorical
*CFR*	Anonymized CFR number associated with the vessel	Categorical
*Vessel_Type*	The vessel type registered in the EU FR data	Categorical
*Main fishing gear*	The registration of the main fishing gear used by the vessel in the EU FR data	Categorical
LOA*	Length overall of the vessel with added random noise (meters)	Float
LBP*	Length between perpendiculars of the vessel with added random noise (meters)	Float
Tonnage GT*	Gross tonnage of the vessel with added random noise	Float
Other tonnage*	Other tonnage information related to the vessel (Tonnes)	Float
Power of main engine*	The power of the main engine of the vessel with added random noise (kW)	Float
Power of auxiliary engine*	The power of the auxiliary engine of the vessel (kW)	Float
*Hull material*	The material used for the hull of the vessel	Categorical
Date of entry into service*	The date when the vessel entered into service with added random noise	Integer
Year of construction*	The year when the vessel was constructed with added random noise	Integer
*real_local*	Code associated with the vessel’s registration Local	Categorical
*Local_Name*	The name of the location where the vessel is registered (only for Portuguese vessels)	Categorical
*I–XIV* variables	Indicator variables for different types of infractions	Binary
*number_infracs*	The number of infractions registered for the vessel	Integer
*Year*	The year of the inspection	Integer
*Month*	The month of the inspection	Integer
*Day*	The day of the inspection	Integer
*Period*	The period of the day of the inspection	Categorical
*NUTS II_Code*	NUTS II code representing the region where the vessel is registered (only for Portuguese vessels)	Categorical

**Table 7:** Comparison of the means and covariances of the perturbed variables calculated from the original and the protected datasets.

Variable	Original Mean	Protected Mean	Original Variance	Protected Variance
*LOA*	11.467893	11.460244	42.572205	46.282576
*LBP*	13.031482	13.059856	35.808518	39.730192
*Tonnage GT*	29.095551	36.692501	3180.355289	2950.528324
*Power of main engine*	113.076253	119.412629	18284.676221	18666.126755
*Date of entry into service*	1993.418422	1993.402055	260.613887	285.513118
*Year of construction*	1992.816798	1992.797640	279.031780	305.537906

**Table 8:** Comparison between *Pearson* and *Kendall* correlations between the number of infractions detected in a vessel and the variables where noise addition was applied in both original and protected data.

Variable	Number of Infractions			
	*Pearson*		*Kendall*	
	Original data	Protected data	Original data	Protected data
*LOA*	0.183503	0.175143	0.174021	0.158154
*LBP*	0.110379	0.098568	0.118285	0.103662
*Tonnage GT*	0.120172	0.105599	0.178250	0.104214
*Power of main engine*	0.154544	0.143508	0.173864	0.141693
*Date of entry into service*	−0.006641	−0.006642	−0.016354	−0.018762
*Year of construction*	−0.003901	−0.001887	−0.019317	−0.019739

## Technical Validation

In this section, a validation of the dataset’s quality will be performed. This analysis is even more critical in a dataset where some techniques were applied to ensure anonymization, which is essential to ensure that the final dataset^8^ presents the needed accuracy.

### Quality Assessment

During the process of disclosure control in datasets, it is inevitable that some level of data quality may be compromised. However, our objective in the transformations performed was to strike a balance between preserving the best possible data quality and ensuring the protection of direct identification disclosure.

While some inherent risk is still involved, it is essential to note that it is not absolute or 100% for all vessels. The level of risk varies depending on the specific variables and their transformations. For vessels that have never recorded a presumable infraction, the risk of direct identification is limited due to the lack of sensitive information associated with them.

Some variables were identified as non-essential for the analysis, and their removal helped preserve the dataset’s confidentiality. The following variables have been removed from the dataset as they were deemed to be not very relevant information about the vessel and posed a high risk of disclosure:

- *AIS indicator*;
- *ERS indicator*;
- *Hour*;
- *IRCS indicator*;
- *License indicator*;
- *Place of registration*;
- *Public aid*;
- *Segment*;
- *Subsidiary fishing gear 1*;
- *Subsidiary fishing gear 2*;
- *Subsidiary fishing gear 3*;
- *Subsidiary fishing gear 4*;
- *Subsidiary fishing gear 5*;
- *VMS indicator*.

**Fig. 5:**
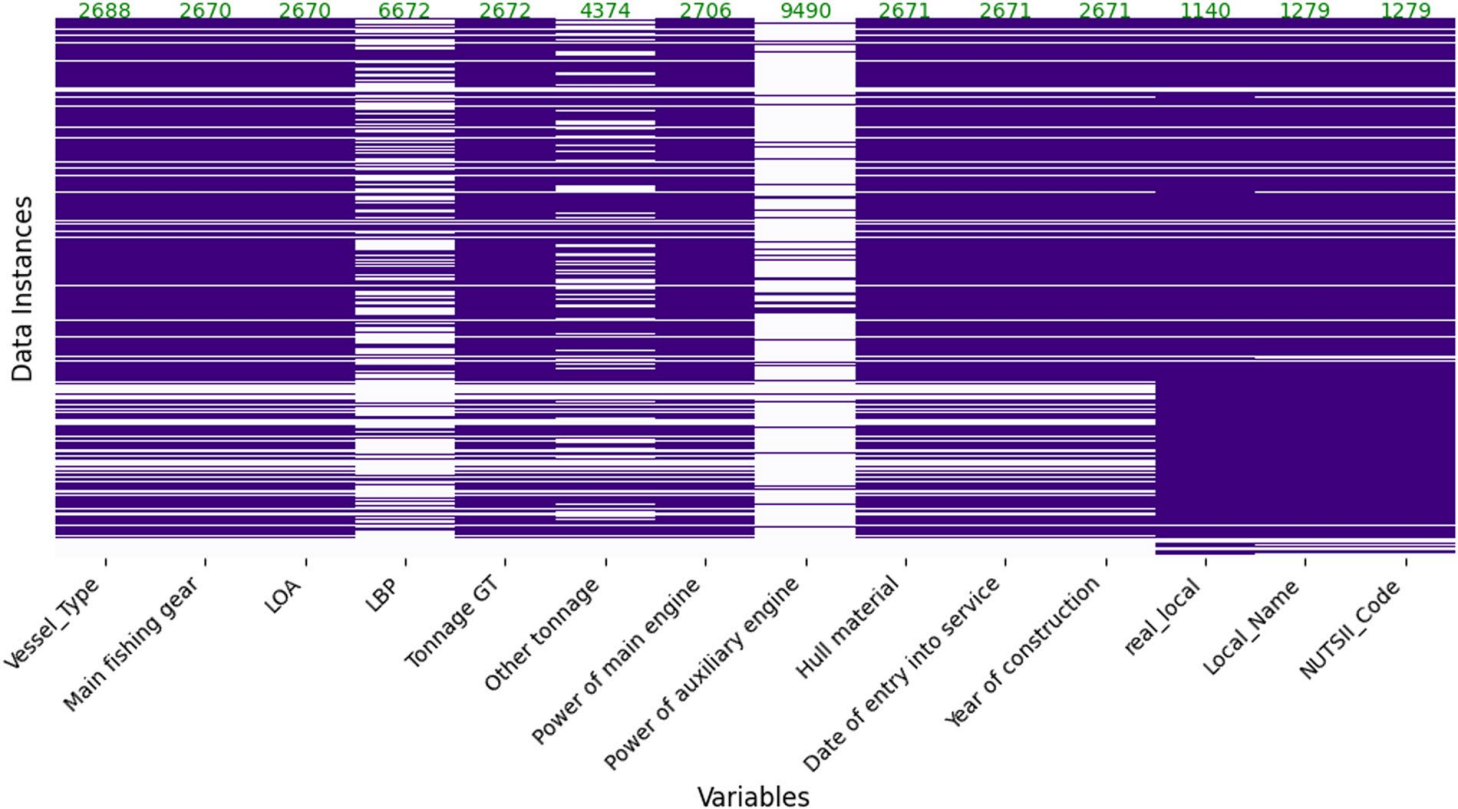
Annotated heatmap displaying missing values in the variables with missing data in the protected dataset. The white color indicates the presence of missing values.

Some variables in the protected dataset do not have values due to a lack of available details for data merging. Additionally, very few variables may have missing values as part of disclosure control measures. Figure 5 provides an overview of the extent of missing data by displaying a heatmap specifically for variables with non-zero missing values. Notably, variables such as *real_local*, *Local_Name*, and *NUTSII_Code* show relatively fewer missing values, indicating successful merging of vessel data from various sources. Most of the remaining missing data is attributed to the absence of records in the EU FR data for some variables.

The rounding transformation applied to the variables can be considered a *perturbative* or *non-perturbative* anonymization method, depending on the level of rounding and the range of the transformed data^14,19^. However, in this specific case, the rounding transformation will not significantly impact the utility or information content of the variables. Rounding to the nearest integer or decimal place merely adjusts the values to a broader level of precision without altering the overall distribution or patterns in the data. As a result, further evaluation of these variables’ utility is unnecessary after the rounding transformation^20^.

The same applies to the variables altered by local suppression^12^. The removal of specific values through local suppression was done to protect sensitive information and reduce the risk of identification. However, since the number of suppressed values is relatively small, it does not significantly impact the utility or information content of the data.

Concerning the imputed values, their number is minimal, and they were used to replace missing or undisclosed values in the dataset. These imputations’ impact on the data is minimal, so they can be considered *non-perturbative*.

**Fig. 6:**
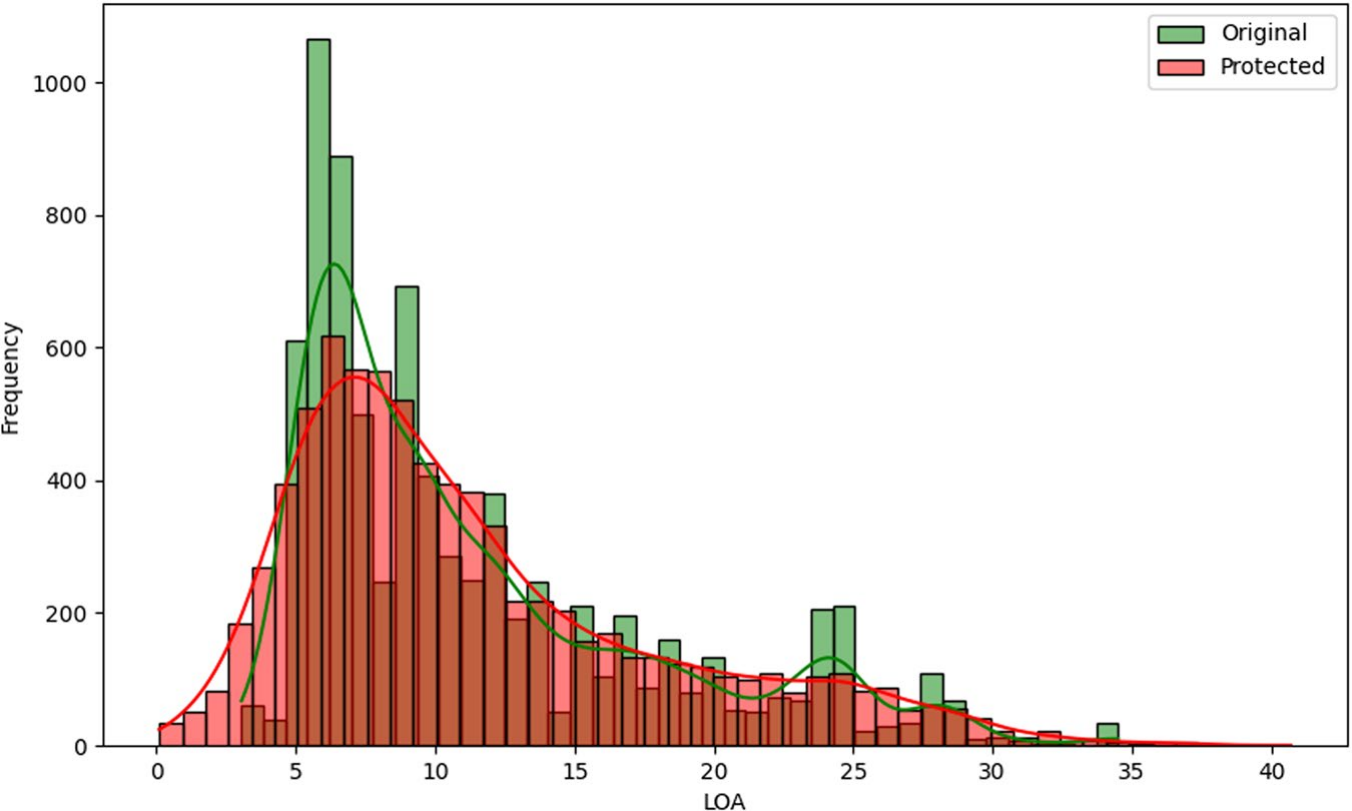
Histogram comparing the distribution of *LOA* between the original and protected datasets with the estimated kernel probability density.

**Fig. 7:**
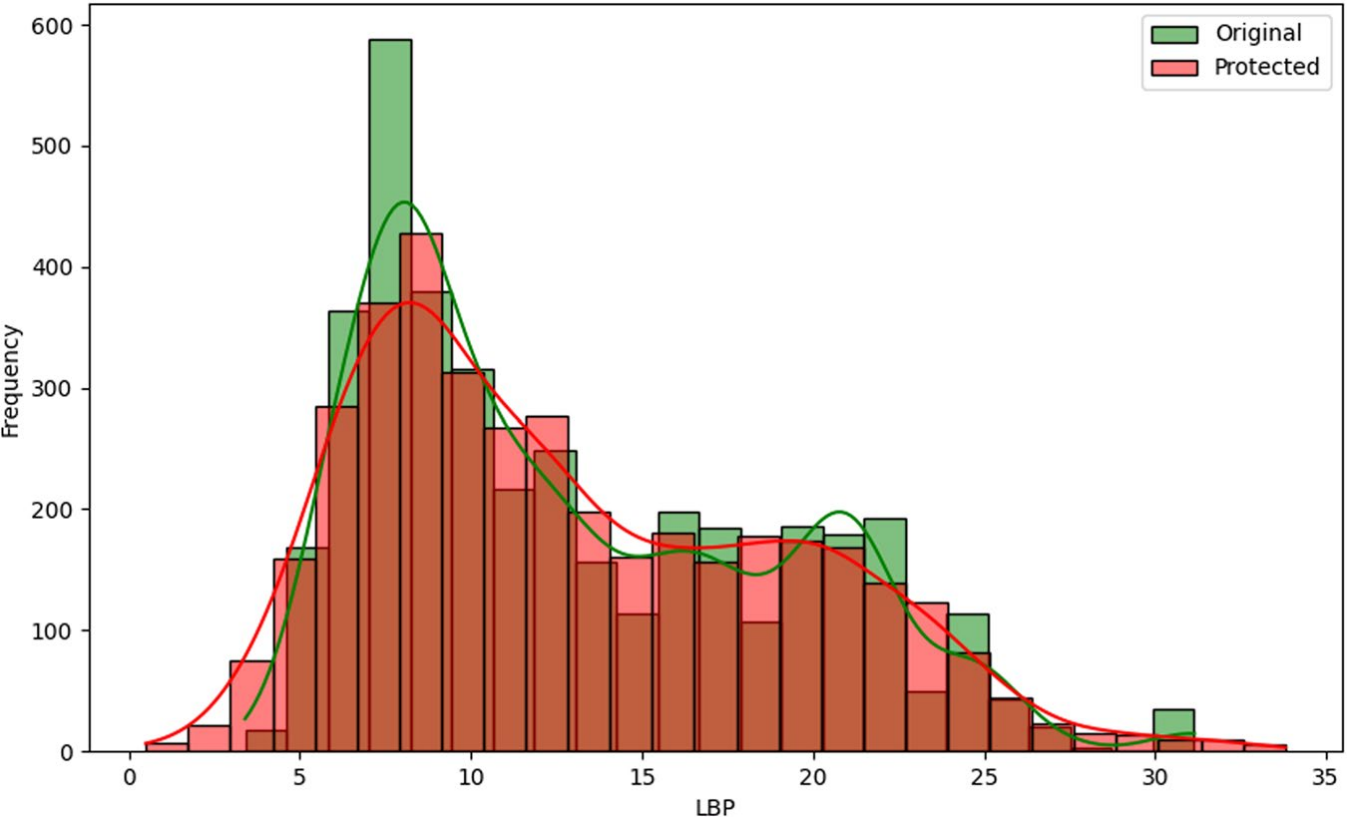
Histogram comparing the distribution of *LBP* between the original and protected datasets with the estimated kernel probability density.

**Fig. 8:**
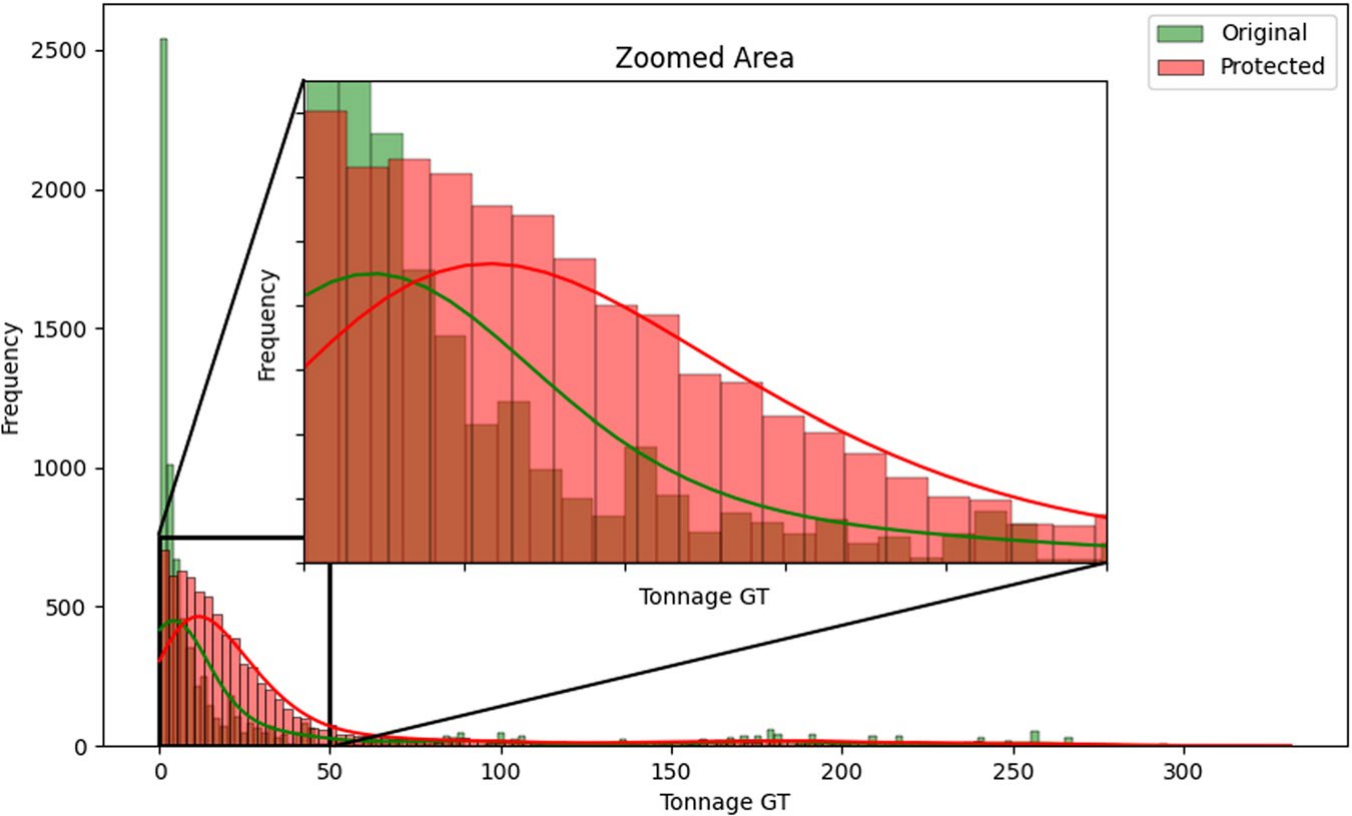
Histogram comparing the distribution of *Tonnage GT* between the original and protected datasets with the estimated kernel probability density.

**Fig. 9:**
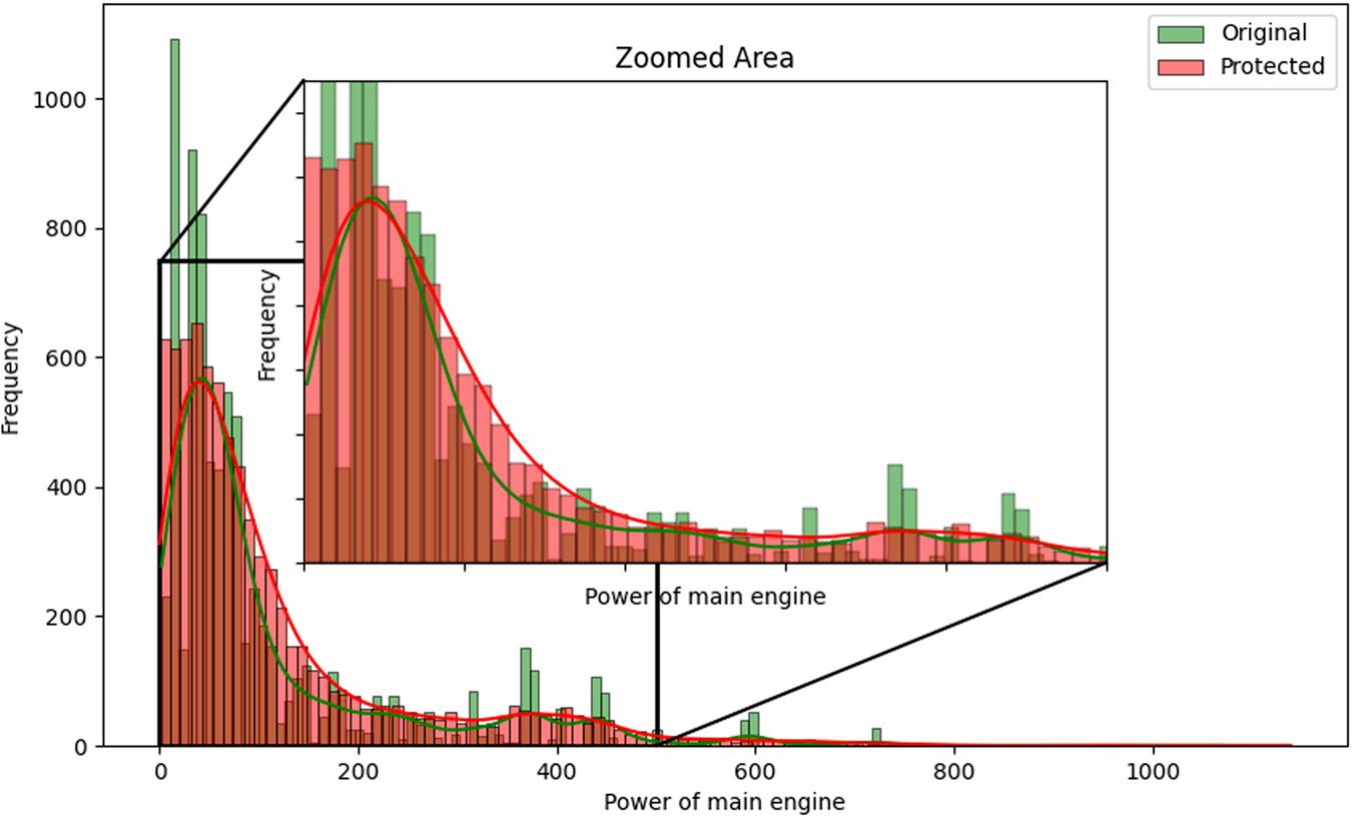
Histogram comparing the distribution of *Power of main engine* between the original and protected datasets with the estimated kernel probability density.

**Fig. 10:**
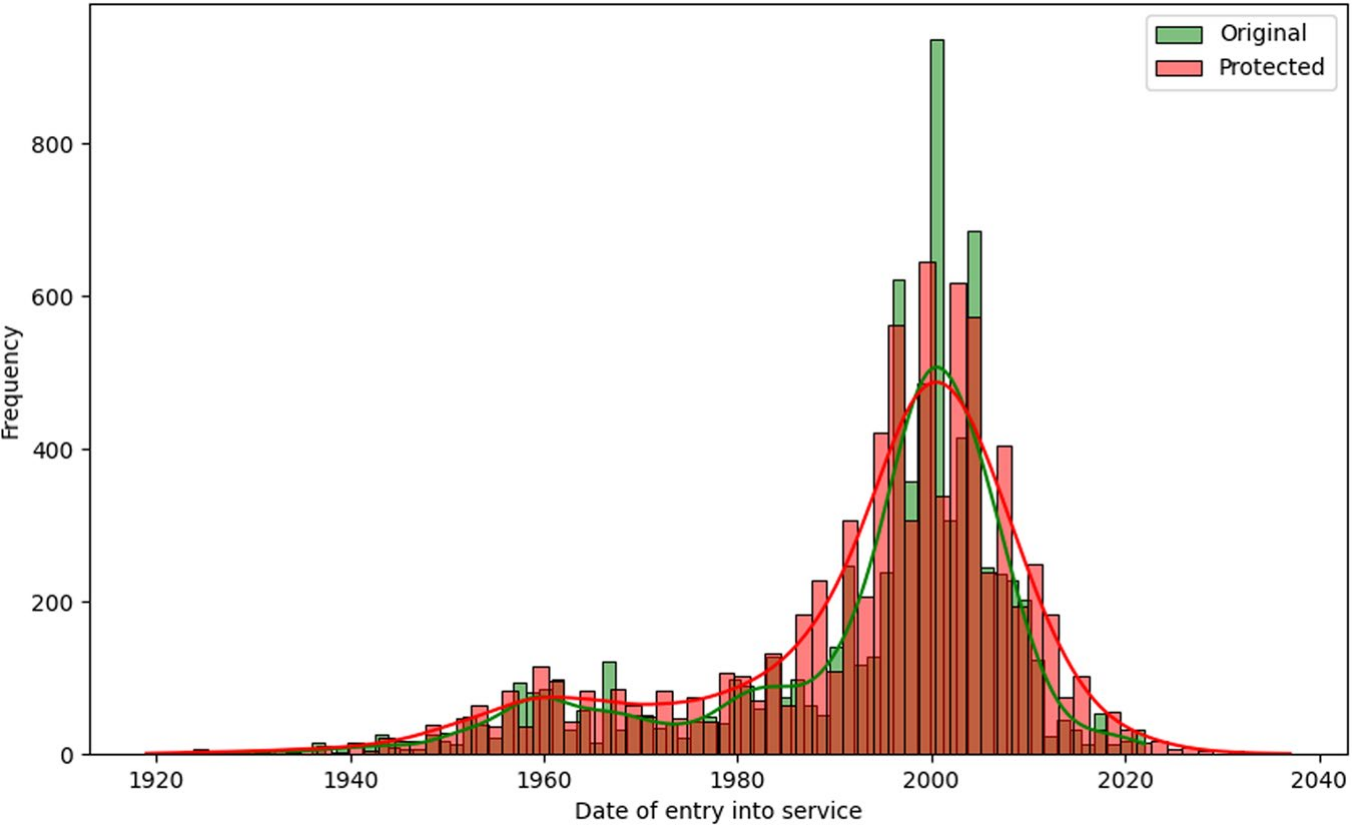
Histogram comparing the distribution of *Date of entry into service* between the original and protected datasets with the estimated kernel probability density.

**Fig. 11:**
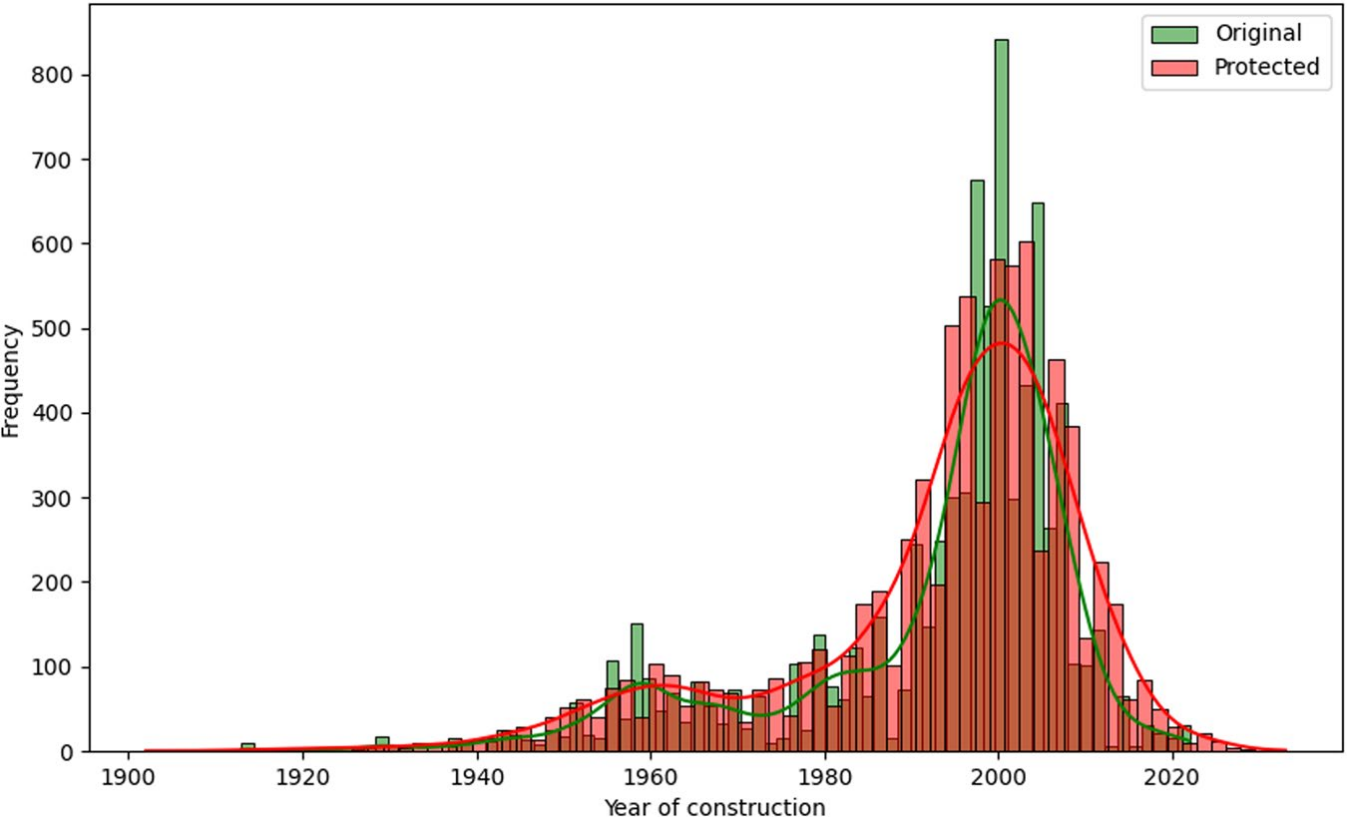
Histogram comparing the distribution of *Year of construction* between the original and protected datasets with the estimated kernel probability density.

Some utility measures can be performed regarding the variables perturbed by random noise, where all records were changed. One of the first analyses can be visual^13,20^. The histograms comparing the distributions of the variables *LOA*, *LBP*, *Tonnage GT*, *Power of main engine*, *Date of entry into service*, and *Year of construction* between the original and protected datasets, are represented in Figs. 6, 7, 8, 9, 10, and 11.

We may observe from the figures’ analysis that the perturbed variables have distributions that closely resemble the original ones. However, due to the addition of noise, the density of the perturbed variables appears slightly smoother compared to the original data. This is because the noise added follows a normal distribution, resulting in a slight smoothing effect on the distribution of the variables, which was expected. The smoothness introduced by adding noise to the perturbed variables can help improve data quality by reducing the influence of outliers and enhancing data integrity. However, it may also result in losing fine-grained detail and introduce potential bias.

Another aspect to note is the distribution of the variables *Tonnage GT* and *Power of main engine*, as illustrated in Figs. 8 and 9. When observing their distributions in smaller intervals closer to zero, these variables exhibit a slight rightward displacement in their distributions. This displacement was expected, as we recoded the values to be non-zero and non-negative, assigning the minimum recorded value when zero and the absolute value when negative. However, it is essential to highlight that this adjustment did not significantly alter the overall distribution of the variables.

Another way to assess data quality is by comparing variable statistics between the protected and original datasets, such as means and variances. This comparison allows us to evaluate how well the data anonymization techniques have preserved the statistical properties of the original data^21^. In Table 7, one may see the mean and variance of the perturbed variables calculated from the original and protected datasets.

With careful observation of the statistics described in Table 7, it is clear that the variables with the highest difference have larger variances, as the noise addition perturbation incorporated these into its calculation. However, it is essential to note that the differences between the original and protected dataset statistics are relatively small overall if we compare the normalized distance between the original and protected data as:

4 $${d}_{i}=\frac{| {\bar{x}}_{i}-{\bar{x}}_{i}^{* }| }{{\sigma }_{i}}$$

where ${\bar{x}}_{i}$ and ${\bar{x}}_{i}^{* }$ are the original and protected variable means and *σ*_*i*_ is the original standard deviation. The values for variables *LOA*, *LBP*, *Tonnage GT*, *Power of main engine*, *Date of entry into service*, and *Year of construction*, were, respectively, 0.001172, 0.004742, 0.134710, 0.046859, 0.001014, and 0.001147. We can notice that all values are very close to zero, indicating that the data anonymization techniques have successfully preserved the original data’s essential characteristics while introducing privacy protection.

One interesting measure/statistic to compare is the alteration in the correlation between these variables and the number of infractions recorded. The *Pearson* correlation measures the linear relationship between variables, and thus, we can observe its change in Table 8. However, since the perturbed variables have outliers, it is also essential to consider *Kendall*’s correlation. *Kendall*’s correlation is a robust coefficient that will evaluate the strength and direction of the ordinal association between pairs of variables since we may have non-linear relationships and the data contains outliers^22^.

Based on the correlation values presented in Table 8, we may observe that the original and protected datasets have similar correlation patterns between the numeric variables and the categorical variable *number_infracs*. The differences in correlation values between the two datasets are minimal. Therefore, in terms of correlation, the original and protected datasets provide identical information and do not show significant differences. By including *Kendall*’s correlation, we gained additional insights into the relationship between the perturbed variables and the number of infractions before and after protection.

**Fig. 12:**
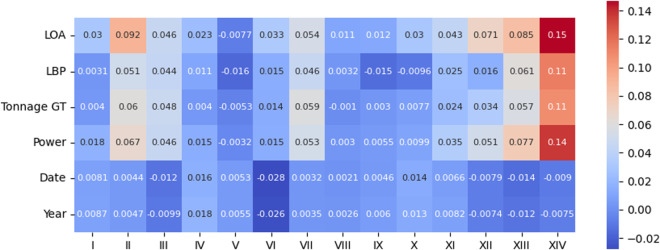
*Pearson* correlation between the perturbed variables and type of infractions on the original data.

**Fig. 13:**
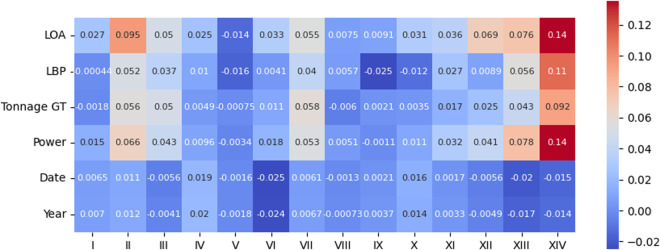
*Pearson* correlation between the perturbed variables and type of infractions on the protected data.

**Fig. 14:**
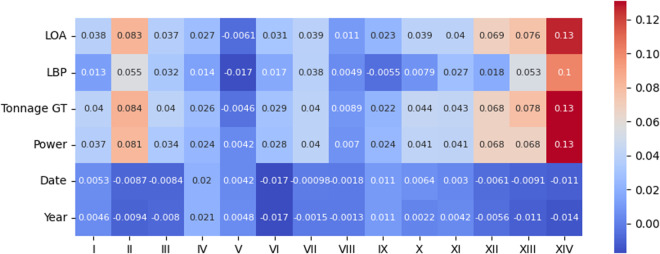
*Kendall* correlation between the perturbed variables and type of infractions on the original data.

**Fig. 15:**
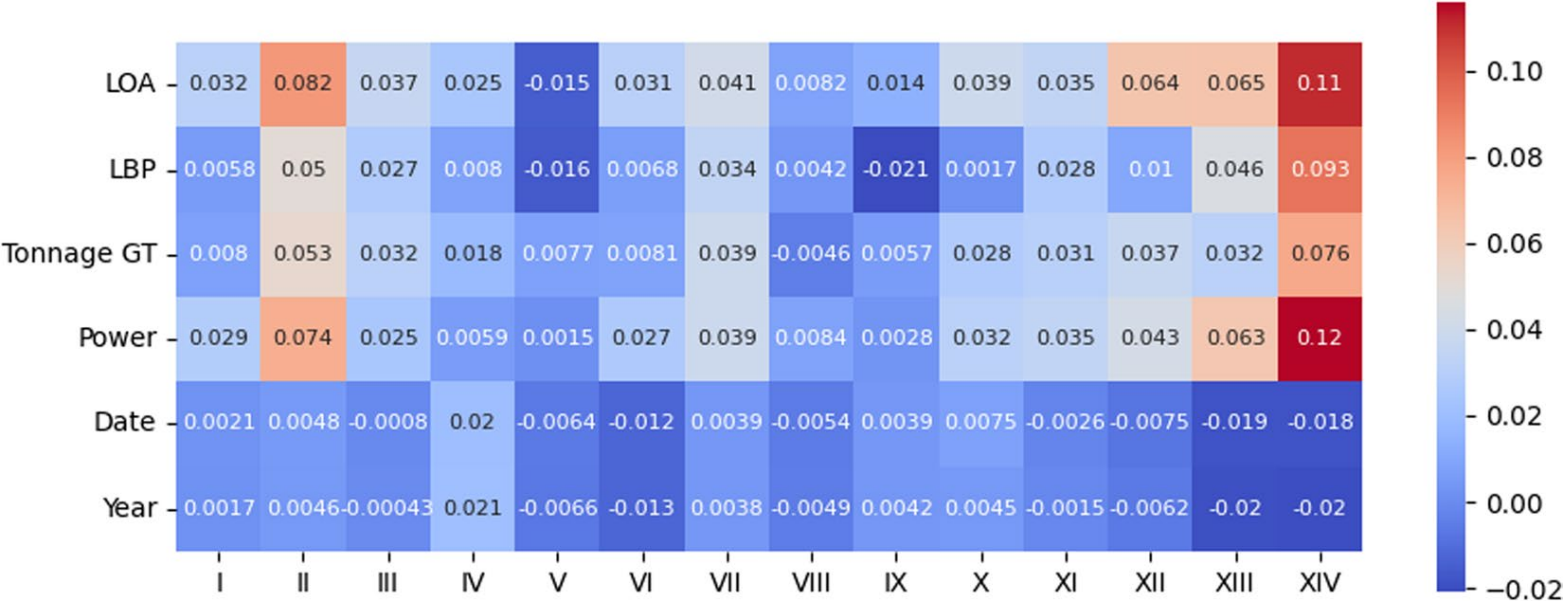
*Kendall* correlation between the perturbed variables and type of infractions on the protected data.

We may also analyze the correlation between the perturbed variables and the presence of each of the 14 infractions, the motivating variable for constructing this dataset. For that reason, we present both *Pearson* and *Kendall* correlation coefficient matrices in Figs. 12, 13, 14, and 15.

**Fig. 16:**
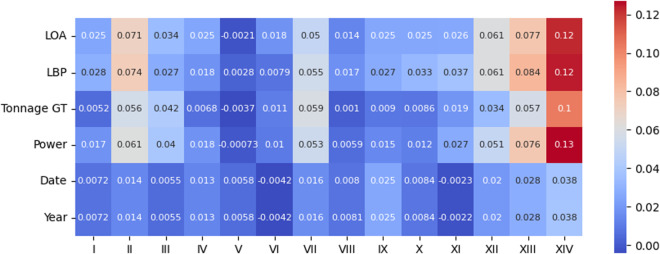
*Point-biserial* correlation between the perturbed variables and type of infractions on the original data.

**Fig. 17:**
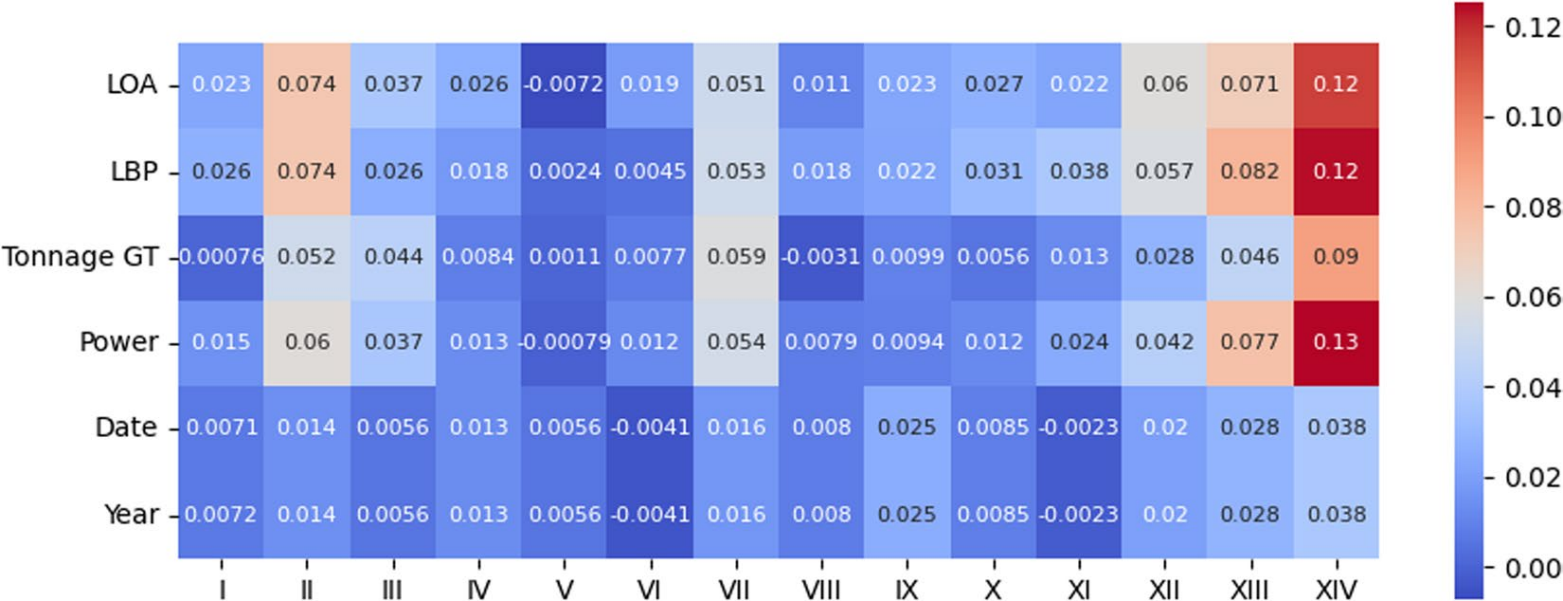
*Point-biserial* correlation between the perturbed variables and type of infractions on the protected data.

After analyzing the figures, we can clearly state that the correlation matrices present similar values overall. However, it is essential to note that, in this case, we want to quantify the relationship between a continuous variable and a binary variable. The *Point-biserial* correlation^23,24^ was designed for that purpose, and with it, we can effectively capture and assess the degree of association between the perturbed variables and the occurrence of different infractions. Figures 16 and 17 illustrate the *Point-biserial* correlation between the perturbed variables and the occurrence of different infractions. These figures analysis provide a clear depiction of the strength and direction of the association between the continuous perturbed variables and the binary infraction types, and again the values do not significantly differ.

Overall, the quality assessment of the data revealed that the disclosed data maintained its usefulness for analysis and research purposes. The applied anonymization techniques and transformations ensured the protection of vessel identification while preserving the majority of the integrity and utility of the data.

## Usage Notes

The original dataset underwent a partial transformation to safeguard vessel identification, resulting in a modified dataset that does not directly represent the original data. However, it is essential to note that the level of disturbance introduced to the data was minimal, as shown in the previous section. As a result, users can utilize this perturbed dataset as a substitute for the original data when studying patterns and behaviors.

Independent errors were added to the variables using a noise addition technique to protect privacy and confidentiality. From a statistical standpoint, this method can be conceptualized as generating a standard errors-in-variables problem. This allows for a better understanding of the statistical properties and implications associated with the perturbed dataset. Using such techniques, researchers can still extract meaningful insights and draw valid conclusions from the perturbed data while maintaining the necessary privacy protections.

The original dataset was partially transformed to protect vessel identification. Thus, the available dataset does not represent the original data. Despite that fact, the level of disturbance in the data was insignificant, as shown in the previous section. Thus, any user may use it if it was the original data to study patterns and behaviors.

For linear regression models, the effects of measurement errors on the properties of linear estimators have been extensively studied in the literature on errors-in-variables models. Researchers such as *Fuller*^25^, *Lechner and Pohlmeier*^26^, and others have compared different masking procedures, including masking by noise addition, and investigated their implications for linear regression models. Although errors-in-variables in nonlinear models have not been extensively studied in the literature, some notable works address specific aspects of measurement errors in nonlinear settings. For instance, Schennach^27^ and Hong and Tamer^28^ delve into this topic and offer valuable insights. Additionally, researchers interested in further exploring these areas can consult the works of Schennach^29^ and Nakamura^30^, which provide in-depth discussions and corrected methodologies for dealing with measurement errors in nonlinear models.

In the context of our dataset, it is good to consider the perturbation’s impact on the variables’ statistical properties. While the perturbed data may introduce measurement errors and affect the accuracy of estimators, techniques developed in the errors-in-variables literature can offer valuable insights and guidance for analyzing and interpreting this data. Nevertheless, using the overall dataset as an original dataset is also advised since just some variables were transformed in the disclosure process.

## Data Availability

The code used for the extraction, translation, pre-processing, and protection of the vessel identification is available in a GitHub repository (https://github.com/ricardomourarpm/Fishery_Inspection_PT_2017_23). To run the provided code, it is possible to run it locally using Python in a Jupyter Notebook or even use the Anaconda Distribution, or you can use it directly online using, e.g., the Google Colaboratory (https://colab.research.google.com/). The Anaconda Distribution (https://jupyter.org/install.html) is an excellent choice for scientific computing and data science as it includes Python, Jupyter Notebook, and other commonly used packages. One must use synthetically generated or protected data to run the codes since the original dataset cannot be shared for privacy and confidentiality reasons. Moreover, some values/variable vectors in files *4_1_k_identifiers_analysis.py* and *5_Anonimization.py* are not real (https://github.com/ricardomourarpm/Fishery_Inspection_PT_2017_23), as they should be withheld from the general public to ensure data protection and privacy.
